# Contribution of Clinical Metagenomics to the Diagnosis of Bone and Joint Infections

**DOI:** 10.3389/fmicb.2022.863777

**Published:** 2022-04-21

**Authors:** Camille d’Humières, Nadia Gaïa, Signara Gueye, Victoire de Lastours, Véronique Leflon-Guibout, Naouale Maataoui, Marion Duprilot, Marie Lecronier, Marc-Antoine Rousseau, Naura Gamany, François-Xavier Lescure, Olivia Senard, Laurène Deconinck, Marion Dollat, Valentina Isernia, Anne-Claire Le Hur, Marie Petitjean, Anissa Nazimoudine, Sylvie Le Gac, Solaya Chalal, Stéphanie Ferreira, Vladimir Lazarevic, Ghislaine Guigon, Gaspard Gervasi, Laurence Armand-Lefèvre, Jacques Schrenzel, Etienne Ruppé

**Affiliations:** ^1^AP-HP, Hôpital Bichat, Service de Bactériologie, Paris, France; ^2^INSERM, Université de Paris Cité, IAME, Paris, France; ^3^Laboratoire de Recherche Génomique, Hôpitaux Universitaires de Genève, Genève, Switzerland; ^4^AP-HP, Hôpital Beaujon, Service de Médecine Interne, Paris, France; ^5^AP-HP, Hôpital Beaujon, Laboratoire de Bactériologie, Paris, France; ^6^AP-HP, Hôpital Beaujon, Service de Chirurgie Orthopédique, Paris, France; ^7^AP-HP, Hôpital Bichat, Service de Maladies Infectieuses, Site Bichat, Paris, France; ^8^AP-HP, Hôpital Bichat, Département d’Epidémiologie Biostatistique et Recherche Clinique, Paris, France; ^9^Genoscreen, Lille, France; ^10^bioMérieux SA, Marcy-l’Etoile, France

**Keywords:** clinical metagenomics, bone and joint infections, diagnosis, Illumina, 16S rDNA gene analysis

## Abstract

Bone and joint infections (BJIs) are complex infections that require precise microbiological documentation to optimize antibiotic therapy. Currently, diagnosis is based on microbiological culture, sometimes complemented by amplification and sequencing of the 16S rDNA gene. Clinical metagenomics (CMg), that is, the sequencing of the entire nucleic acids in a sample, was previously shown to identify bacteria not detected by conventional methods, but its actual contribution to the diagnosis remains to be assessed, especially with regard to 16S rDNA sequencing. In the present study, we tested the performance of CMg in 34 patients (94 samples) with suspected BJIs, as compared to culture and 16S rDNA sequencing. A total of 94 samples from 34 patients with suspicion of BJIs, recruited from two sites, were analyzed by (i) conventional culture, (ii) 16S rDNA sequencing (Sanger method), and (iii) CMg (Illumina Technology). Two negative controls were also sequenced by CMg for contamination assessment. Based on the sequencing results of negative controls, 414 out of 539 (76.7%) bacterial species detected by CMg were considered as contaminants and 125 (23.2%) as truly present. For monomicrobial infections (13 patients), the sensitivity of CMg was 83.3% as compared to culture, and 100% as compared to 16S rDNA. For polymicrobial infections (13 patients), the sensitivity of CMg was 50% compared to culture, and 100% compared to 16S rDNA. For samples negative in culture (8 patients, 21 samples), CMg detected 11 bacteria in 10 samples from 5 different patients. In 5/34 patients, CMg brought a microbiological diagnosis where conventional methods failed, and in 16/34 patients, CMg provided additional information. Finally, 99 antibiotic resistance genes were detected in 24 patients (56 samples). Provided sufficient genome coverage (87.5%), a correct inference of antibiotic susceptibility was achieved in 8/8 bacteria (100%). In conclusion, our study demonstrated that the CMg provides complementary and potentially valuable data to conventional methods of BJIs diagnosis.

## Introduction

Bone and joint infections (BJIs) are serious infections affecting a growing number of patients. BJIs generate a significant economic burden, in addition to the related morbidity and mortality (Kurtz et al., 2012). From the clinician’s perspective, BJIs are complex infections that require precise microbiological documentation to tailor the antimicrobial therapy that is usually necessary for several weeks (Osmon et al., 2013). Nonetheless, such documentation is challenging with regard to the diversity of microorganisms which may be involved in BJIs: aerobic/anaerobic bacteria (Ruppé et al., 2017), fastidious bacteria (e.g., *Mycoplasma*; Thoendel et al., 2017), fungi (Bariteau et al., 2014), and/or mycobacteria (Hogan et al., 2019). Current evidence reports that in approximately 80% of cases, a single causative agent is found (referred to as monomicrobial infections), while in up to 10% of cases, more than one microorganism is found (polymicrobial infections) (Tande and Patel, 2014; Tan et al., 2016). Meanwhile, the frequency of culture-negative BJIs varies from 5 to 35% (Tande and Patel, 2014).

Currently, routine BJIs diagnosis strongly relies on microbiological culture. Given the diversity of microorganisms potentially expected, different sets of culture conditions, including various media, incubation atmospheres, and incubation time, are used. When culture results are negative while the suspicion of BJIs remains high, molecular methods such as the amplification and sequencing of the 16S rRNA-encoding gene (Sanger sequencing) can be used. 16S rDNA sequencing can identify fastidious-growing bacteria and bacteria which cannot grow because they were killed by a previous antibiotic exposure (Fida et al., 2021), but it performs poorly in polymicrobial samples when more than one microorganism must be identified. Moreover, 16S rDNA sequencing does not provide any information related to antimicrobial resistance.

Clinical metagenomics (CMg) refers to the metagenomic sequencing of nucleic acids extracted from a sample that may contain mixed populations of microorganisms to obtain information of clinical relevance (Chiu and Miller, 2019). CMg can potentially (*i*) identify which microorganisms are present, (*ii*) estimate their relative proportions, and (*iii*) provide information related to their susceptibility to antimicrobials. CMg was first reported in 2014 with the sequencing of a cerebrospinal fluid sample obtained from a 14-year-old boy suffering from neuroleptospirosis (Wilson et al., 2014). This technique has since proven relevant in a vast array of clinical situations (Chiu and Miller, 2019). In suspected bacterial infections, the main advantage of CMg over conventional methods is its capacity to identify (i) bacteria not detected by routine methods, (ii) antimicrobial resistance genetic determinants, and (iii) other genes of interest such as virulence factors–encoding genes. Six studies (Ruppé et al., 2017; Street et al., 2017; Ivy et al., 2018; Sanderson et al., 2018; Thoendel et al., 2018; Zhao et al., 2020) investigated the performance of CMg in comparison to culture-based methods in diagnosing BJIs. The sensitivity of CMg varied from 58% at the species level in polymicrobial samples (Ruppé et al., 2017) to 100% in monomicrobial samples (Thoendel et al., 2018). A recent study showed a 79% sensitivity of CMg compared to the combination culture/16S rDNA sequencing for the microbiology diagnosis of 182 body fluids (including 21 joint fluids; Gu et al., 2021). However, data concerning the comparison between CMg and the combination culture-16S rDNA sequencing are lacking.

In addition to the identification of the microorganisms, CMg can be leveraged to infer their antimicrobial susceptibility profile from metagenomic data. In a previous study from our group (Ruppé et al., 2017), we searched for antibiotic resistance genes and mutations associated with resistance (such as mutations in topoisomerases-encoding genes associated with quinolone resistance) and tried to infer a global (sample-level) antibiotic susceptibility phenotype. A correct antibiotic susceptibility level could be inferred in 94 and 77% of monomicrobial and polymicrobial samples, respectively (Ruppé et al., 2017).

A major issue in CMg concerns dealing with human DNA. In samples taken from various body locations, recovery of microbial DNA competes with that of human DNA. The competition is biased, however, in that the human DNA is approximately 1,000-fold longer than the size of an average bacterial genome. Hence, solutions to deplete the human DNA before sequencing have been proposed, such as the use of specific eukaryotic cell lysis before DNA extraction followed by the removal of free DNA (Ruppé et al., 2017; Charalampous et al., 2019). Nonetheless, this strategy is compromised by freezing–thawing cycles, which promote the lysis of microbial cells. In a previous study (Ruppé et al., 2017), we indeed obtained a minimal amount of bacterial DNA required for sequencing for only 24 out of 179 BJI samples, which had been under freezing–thawing cycles.

In this study, we aimed to compare CMg to the combination of culture and 16S rDNA sequencing applied to non-frozen samples. An additional goal was to sequence several samples per patient and to consider the overall results to assess the potential added value of CMg over conventional methods.

## Materials and Methods

### Sample Collection

**FIGURE 1:**
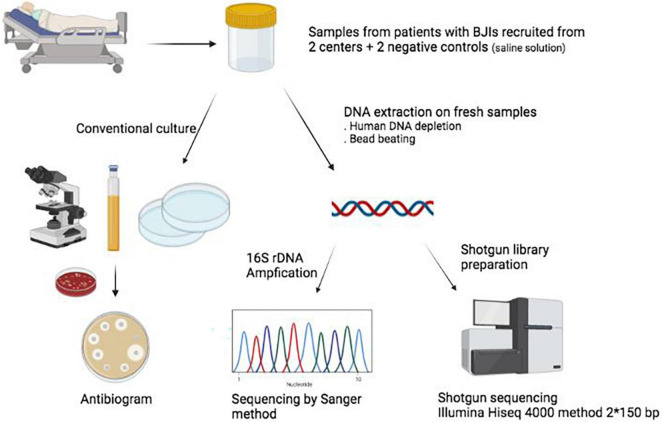
Summary of the study. BJIs, bone and joint infections. Figure created with biorender.com.

Samples from patients undergoing surgery for BJI suspicion were collected from November 2018 to June 2019 in two hospitals in the Paris area (France): Bichat-Claude Bernard Hospital (Paris) and Beaujon Hospital (Clichy). According to current recommendations in BJI surgery, more than one sample (typically 3–5) per patient was collected and sent to the respective microbiology laboratories. For this study, we also included two negative controls (one in each center), that is, saline solution following the very same processing as the samples but without any biological material (Figure 1). This project obtained clearance from the ethical committee [Comité d’Evaluation de l’Ethique des projets de Recherche Biomédicale (CEERB) “Paris Nord”; IRB 00006477]. 16S rDNA and metagenomic sequencing were performed at distance from the inclusion of patients, and their results were not disclosed to the clinicians in charge of the patient.

### Culture Methods

The following describes standard cultivation practices in the two bacteriology laboratories in which the present study was conducted. In Bichat Hospital: non-liquid samples (bone, tissue) were homogenized by bead-beating using a sterile tube (Labomoderne, Gennevilliers, France) at 6,000 rpm for 3 min. Then, they were plated onto: (i) two sheep blood agar Columbia (COS) plates (bioMérieux SA, Marcy l’Etoile, France) incubated, respectively, under aerobic and anaerobic conditions at 36 ± 1°C, (ii) one chocolate polyvitex agar plate (bioMérieux SA, Marcy l’Etoile, France) kept under a 5% CO_2_-enriched aerobic atmosphere at 36 ± 1°C, (iii) one buffered glucose broth (bioMérieux SA, Marcy l’Etoile, France) incubated 2 days at 36 ± 1°C, and (iv) one semi-liquid Schaedler broth (bioMérieux SA) incubated 10 days at 36 ± 1°C. In Beaujon Hospital: tissue samples were disrupted with bead-beating (Retsch MM301, Verder, Eragny, Oise, France) while bone and material samples were sonicated 4 min (B200 Ultra sonic cleaner, Branson). Then, they were plated onto (i) two sheep blood agar Columbia (COS) plates (bioMérieux SA, Marcy l’Etoile, France) incubated, respectively, under aerobic and anaerobic conditions at 36 ± 1°C, (ii) one chocolate polyvitex agar plate (bioMérieux SA, Marcy l’Etoile, France) kept under a 5% CO_2_-enriched aerobic atmosphere at 36 ± 1°C and (iii) two BACT/ALERT vials (aerobic and anaerobic) (bioMérieux SA) incubated in a BACT/ALERT system for 18 days.

For all samples, whenever cultures were positive, microorganisms were identified using matrix-assisted laser desorption ionization–time of flight mass spectrometry (MALDI Biotyper, Bruker Daltonics, Bremen, Germany) and antimicrobial susceptibility was tested according to the French Committee for antibiotic susceptibility testing (V2.0 May 2019) guidelines. Samples were considered to be monomicrobial when only one bacterium had grown in culture and polymicrobial when more than one bacterium had grown.

### DNA Extraction and Sequencing Methodology

DNA was extracted from fresh samples (with no prior freezing/thawing step) using the Ultra-Deep Microbiome Prep kit (Molzym, Bremen, Germany) according to the manufacturer’s instructions for tissues, then frozen to –80°C. DNA was transferred to GenoScreen (Lille, France) for sequencing. DNA was prepared using the Kit Nextera XT (Illumina, San Diego, CA) with an input of 1 ng, and sequencing was performed on an Illumina HiSeq4000 with 150-base paired-end reads. Samples with a concentration > 0.05 ng/μL were be prepared according to the standard library preparation protocol (with 12 cycles of amplification); those with concentrations < 0.05 ng/μL were prepared according to the library preparation protocol with 20 cycles of amplification. The reads have been deposited as the Meta-prOSpEcT bioproject (PRJNA803614). On the same DNA extract, an amplicon covering the V1–V5 regions of the 16S rRNA–encoding gene was amplified and sequenced (Sanger method) when positive (Figure 1), using the following primers: P8 (AGAGTTTGATCCTGGCTCAG), P535 (GTATTACCGCGGCTGCTGGCAC), 338-1040F (CTC CTACGGGAGGCAG), and 338-1040R (GACACGAGCTGA CGACA).

### Bioinformatic Analyses

For Illumina reads, the Trimmomatic v.0.36 package (Bolger et al., 2014) was used to remove Illumina adapter sequences and to trim low-quality ends of reads at the beginning of any 10-base wide sliding window with an average Phred quality score < 30. Reads whose length was < 90 bases after trimming were discarded. Putative artifactual replicate reads were filtered out using a homemade script that retains the longest sequence among those with identical first hundred nucleotides (or all bases for fragments of 90–99 nt), in either forward or reverse reads. Any forward or reverse reads without their corresponding paired read were discarded. To control for human DNA contamination, we removed all reads assigned to *Homo sapiens* based on the CLARK v.1.2.5 (Ounit et al., 2015) classification (with parameter -m 0) against NCBI/RefSeq (Kitts et al., 2016) reference human genome sequence assembly GRCh38.p7.

The remaining reads were classified at the species levels using MetaPhlAn2 (Truong et al., 2015) with default settings and CLARK (Ounit et al., 2015) (with parameters -m 0 -c 0.8) against a collection of representative and reference prokaryotic genomes from the (NCBI/RefSeq database downloaded on 14 June 2018). In the final analysis, we considered bacteria at the species level with relative abundance higher than 1%.

Reads corresponding to bacterial and archaeal 16S rRNA genes were first selected using USEARCH (Edgar, 2010) 8.1.1861 (-usearch_global -id 0.9 -query_cov 1 -top_hit_only -strand both) against the EzBioCloud 16S database (Yoon et al., 2017) (downloaded on 4 January 2018). The selected reads were then classified by mapping to the EzBioCloud 16S database with USEARCH software (-usearch_global -id 0.95 –*e*-value 0.00001 -strand both -top_hits_only -maxaccept 20). If a read had multiple best hits (identical scores), we retained the match to the organism with higher counts in the sample analyzed.

Acquired antimicrobial resistance genes were searched for with ResFinder (Zankari et al., 2012) v.3.2 (-mp blastn -t 0.9 -l 0.6) and the ResFinder database (as of 1 October 2019). Individual reads were mapped to the ResFinder database using USEARCH (-usearch_global -id 0.90 –*e*-value 0.001 mincols -100 -strand both -top_hits_only -maxaccept 20). Then, reads from samples of the same patient were pooled to increase the *in silico* sequencing depth and assembled using metaSPAdes (Nurk et al., 2017) v.3.12.0 (-k 21,33,55,77,99,127). To calculate genome coverage of a given species (species of interest), we used the QUAST (Gurevich et al., 2013) 5.0.2 “Genome fraction” result obtained by mapping contigs generated by metaSPAdes against the NCBI/RefSeq reference/representative genomic sequence of the species in question.

For Sanger sequences, reads (forward and reverse) were assembled with Sequencher (v 5.4) and the taxonomic assignment was achieved using BLASTn (Altschul et al., 1990) on the nr/nt database with a percentage of homology greater than 99%.

### Statistical Analyses

The sensitivity of CMg and 16S rDNA were calculated compared to culture, at the sample level and the patient level. Only bacterial species considered to be non-contaminants according to the technique tested were included. At the sample level, the sensitivity was computed as follows: the sum of bacterial species numbers found in individual samples by both culture and the tested technique, divided by the sum of bacterial species numbers determined by the culture in these same samples. At the patient level, the sensitivity was computed as follows: the sum of unique bacterial species found in all the samples of a given patient by both culture and the tested technique, divided by the sum of bacterial species numbers determined by the culture in this patient.

## Results

### Sample Description

A total of 35 patients suspected of BJIs were included: 21 from Bichat Hospital and 14 from Beaujon Hospital. A total of 127 samples were collected, with a median number of 3 samples per patient (range 1–7). We considered sequencing a maximum of 3 samples per patient (giving priority to those with the highest amount of DNA) so that 99 were sent for sequencing (CMg and 16S rDNA amplification and sequencing). Four samples were removed because of the low quantity of reads (<100,000 reads), with three of them obtained from the same patient. Hence, CMg performances could be evaluated in 94 samples (34 patients). Characteristics of patients and samples are detailed in Table 1. Culture methods identified polymicrobial infections in 13 patients, monomicrobial infections in 13 patients, and no bacteria in 8 patients. CMg yielded an average number of 1,093,865 paired, high-quality reads per sample (IQR 642,749–1,313,959) (Supplementary Figure 1). After filtering for human reads, an average of 132,073 reads (IQR 9,273–145,536) was obtained, yielding an average human DNA relative abundance of 87% (min 12.8%; max 99.9%). Details of the samples and bacteria identified by culture, 16S rDNA sequencing, and CMg are available in Supplementary Table 1.

**TABLE 1:** Clinical characteristics of the 34 patients included in this study.

	*n* = 34 patients
Age, median [IQR]	66 [47–73]
Male gender (%)	61.8
Number of sample for culture, median [IQR]	3 [3–5]
Number of sample for sequencing, median [IQR]	3 [2–3]
**Body site**	
Knee	3 (8.8%)
Tibia	6 (17.6%)
Hand	3 (8.8%)
Toes	1 (2.9%)
Humerus	1 (2.9%)
Elbow	1 (2.9%)
Radius	1 (2.9%)
Femur	2 (5.9%)
Hip	6 (17.6%)
Spine	8 (23.5%)
Shoulder	2 (5.9%)
**Post-operative infection**	
Yes	28 (82.3%)
With material	21 (75%)
**Delay between surgery and infection**	
NA	6 (17.6%)
<1 month	15 (44.1%)
1–6 months	5 (14.7%)
>6 months	8 (23.5%)

*IQR, interquartile range; NA, not available.*

### Negative Control and Contaminants Management

The two negative controls yielded no bacteria in culture, whereas in CMg, 8 and 19 bacteria were found, respectively, for a total of 22 unique taxa (Supplementary Table 1). Five taxa were found in both samples: *Escherichia coli, Escherichia* unclassified, *Cutibacterium acnes*, *Micrococcus luteus*, and *Propionibacterium granulosum.* We assessed whether these 22 taxa were detected in clinical samples (Supplementary Figure 2A). We observed that *C. acnes, E. coli*, and *Escherichia* unclassified were found in the majority of samples (*C. acnes* 67/94, 71.3%, *E. coli* 49/94, 52.1%, *Escherichia* unclassified 46/94, 48.9%) (Supplementary Figure 2B), with 34 samples (34/94, 36.2%), including the three taxa. Conversely, the simultaneous presence of *C. acnes*, *E. coli*, and *Escherichia* unclassified was not met in 24 samples (24/94, 25.5%). *C. acnes* together with *E. coli* (but not *Escherichia* unclassified) were identified in 24/94 (25.5%) samples while *C. acnes* together with *Escherichia* unclassified (without *E. coli*) were identified in 9/94 (9.6%) samples. Only 9/94 (9.6%) samples had *C. acnes* without *E. coli* or *Escherichia* unclassified. Based on these observations, we used the simultaneous presence of *C. acnes* and *E. coli* or *Escherichia* unclassified and considered as a potential contaminant a taxon when its relative abundance was lower than that of *C. acnes/E. coli* or *C. acnes/Escherichia* unclassified. Next, we aimed at interpreting the results of CMg together with the culture results as it would happen in real life, especially if CMg would be performed at early stages.

### Identification of Microorganisms in Samples

A total of 539 bacteria were identified in the 94 samples. After filtering the bacteria for which the relative abundance was < 1% or lower than that of *C. acnes/E. coli* or *C. acnes/Escherichia* unclassified, 423 (423/539, 78.5%) were considered as potential contaminants. Subsequent interpretation considering culture results added 27 additional bacteria seen as potential contaminants, while 36 initially spotted as potential contaminants were reclassified as non-contaminants. After filtering, a total of 125 bacteria were identified as non-contaminants by CMg.

For 16S rDNA data, 44 bacteria were found in the 94 samples. Among these bacteria, a barley rhizosphere bacterium (*n* = 1) and *Variovorax paradoxus* (*n* = 13) were classified as contaminants (Salter et al., 2014).

### Sensitivity of Clinical Metagenomics Compared to Culture and 16S rDNA Method at the Sample Level

For samples yielding a single bacterium in culture (*n* = 42), the sensitivity at the species level of CMg was 66.6% (28/42). 16S rDNA sequencing identified 19 non-contaminants and had a sensitivity of 40.5% (17/42). Of note, CMg identified 19/19 (100%) bacteria found by 16S rDNA sequencing. Among the 14 cultured bacteria that were not found in CMg, six were recovered only in enrichment broths and five were found in very rare/rare quantities on the plates. Of note in 7 samples, CMg identified another bacterium than the one found in culture. For instance, in patient GM-1–13, *Staphylococcus aureus* was found in enrichment broths of two samples, while in CMg (and 16S rDNA sequencing) a *Streptococcus pyogenes* was found.

For samples in which more than one bacterium had been found in culture (*n* = 21), the total number of bacteria was 62. Among them, 25 (25/62, 40.3%) were detected by CMg. However, CMg identified 42 additional bacteria not detected in culture.

For samples with a negative culture (*n* = 31), CMg detected 19 bacteria not considered as contaminants, while 16S rDNA sequencing identified only three (*Escherichia coli*, *S. aureus*, and *Staphylococcus epidermidis*, also found in CMg).

**FIGURE 2:**
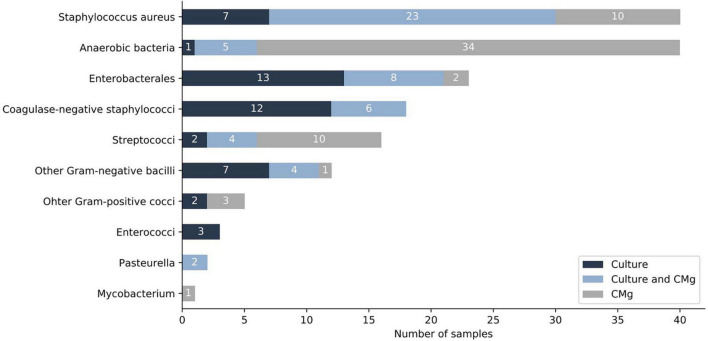
Comparison of culture and CMg for the identification of bacteria (sample level). Dark-blue bars depict bacteria found only by culture. Light-blue bars depict bacteria found in both, culture and CMg. Gray bars depict bacteria found only by CMg. CMg, clinical metagenomics.

When considering all samples (*n* = 94), CMg identified 72 bacteria not found in culture, the most frequent being anaerobic species (*n* = 34) followed by *S. aureus* (*n* = 10) and *S. pyogenes* (*n* = 9) (Figure 2 and Supplementary Table 1).

### Sensitivity of Clinical Metagenomics Compared to Culture and 16S rDNA at the Patient Level

In clinical practice, more than one sample is usually available for interpretation so that the microbiological diagnosis of BJIs is based on the combination of results obtained in all samples. In 47.1% of patients (16/34), 16S rDNA sequencing confirmed the culture result while it brought a new diagnostic in only one case (an *E. coli* found by the 16S rDNA analysis of a sample from a patient where all cultures were negative). CMg has either confirmed the diagnosis made by culture (17/34), established the diagnosis (5/34), completed the microbiological diagnosis (9/34), or challenged the diagnosis (3/34) (Table 2). Taking into account all the samples available for a given patient, for monomicrobial infection (*n* = 12) the sensitivity of CMg compared to culture was 83.3% (10/12). For polymicrobial infections (in which 50 distinct bacteria were identified), the sensitivity of CMg was 50.0% (25/50). The details (per patient) of the bacteria found in culture, in 16S rDNA, and CMg are detailed in Supplementary Table 1.

**TABLE 2:** Patient-level summary of the benefits of 16S rDNA and CMg techniques compared to culture.

	16S rDNA (Sanger sequencing)		Metagenomic sequencing			
Type of infection (culture)	Confirmed the diagnostic	Established the diagnostic	Confirmed the diagnostic	Established the diagnostic	Complemented the diagnostic	Challenged the diagnostic (contamination)
Monomicrobial (*n* = 12)	9	0	10	0	1	0
Polymicrobial (*n* = 14)	0	0	4	0	8	3
Negative (*n* = 8)	7	1	3	5	0	0

*CMg, clinical metagenomics. The method confirmed the diagnostic when the results were in agreement with that of culture. The method established the diagnostic when it identified non-contaminant bacteria while the culture was negative. Metagenomic sequencing complemented the diagnostic when it identified additional, non-contaminant bacteria not found in culture in addition to other bacteria. Metagenomic sequencing challenged the diagnostic when it identified putative contaminant bacteria not found in culture.*

### Antibiotic Resistance Determinants

**FIGURE 3:**
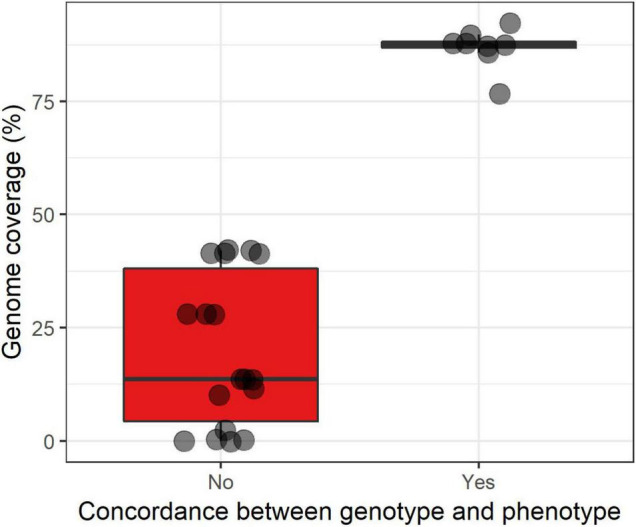
Boxplot superimposed by dots of the correct inference (yes/no) of the antibiotic susceptibility phenotype from metagenomic data according to the estimated genome coverage expressed in percentage. ARG, antibiotic resistance gene. The lower, central, and upper hinges correspond to the first, second (median), and third quartiles. The upper and lower whiskers, respectively, correspond to the higher and lower values at 1.5*IQR from the hinge (where IQR is the inter-quartile range, or distance between the first and third quartiles).

A total of 99 antibiotic resistance genes were detected in 56 samples (from 24 patients). Details are available in Supplementary Table 2 and Supplementary Figure 3. The two most frequent ARGs were an aminoglycoside nucleotidyltransferase (*aadA24*, 16/99, 16.2%) and an aminoglycoside phosphotransferase (*aph3′-IIa*, 14/99, 14.1%). We aimed to assess whether there was a coverage threshold for a correct inference of antimicrobial susceptibility pattern from metagenomic data (Supplementary Table 2). For that, we selected 29 bacteria for which an antibiogram had been performed to connect with the antibiotic resistance determinants found in the metagenome. We were able to link 28 antibiotic resistance determinants to phenotypic traits (e.g., the penicillin resistance gene *bla*Z to penicillin resistance in *S*. *aureus*). Bacteria for which there was a correct prediction (*n* = 8) had a median genome coverage of 87.5% while bacteria for which there has been a failure to detect antibiotic resistance gene (ARG, *n* = 18) (presumably due to the lack of identification of an antibiotic resistance determinant) had a median genome coverage of only 13.6% (Figure 3).

## Discussion

In this study, we assessed the value of CMg in the microbiological diagnosis of BJIs on non-frozen samples, in comparison to conventional culture methods and 16S rDNA sequencing. In 33/34 patients (97.1%), CMg has either confirmed the diagnosis established by culture (17/34) or made/completed/challenged the diagnosis (17/34). Especially, CMg identified 72 bacteria (mainly anaerobic bacteria) not found by culture. However, the sensitivity of CMg was in the range of what had been observed elsewhere, 66.6 and 40.5% in monomicrobial and polymicrobial samples, respectively. This low sensitivity can be explained by various, non-exclusive factors: (i) The difference in inoculum between bacteria can make genomic detection of a subdominant bacterium difficult, (ii) contamination of cultures, (iii) low bacterial inoculum, (iv) too few bacterial reads despite human DNA depletion, and (v) insufficient sequencing depth (Glassing et al., 2016; Leo et al., 2017; Ruppé et al., 2017; Wilson et al., 2019). Nonetheless, we observed that CMg had a sensitivity of 100% compared to 16S rDNA (Sanger method) but allowed completion or diagnostics much more frequently than 16S rDNA. Overall, we found that in the context of BJIs, CMg provided an added value in almost half of the patients, but that it should not replace conventional methods due to low sensitivity. CMg has been considered as a last resort test when all conventional methods have failed to identify any microorganism. Our results argue that, at least in the context of BJIs, CMg could be positioned much earlier, possibly in parallel to conventional methods. An early positioning of CMg would also solve the issue of human DNA depletion during DNA extraction, which is mandatory when dealing with bacterial infections. Indeed, working with fresh samples instead of frozen ones would better preserve bacterial DNA when selective lysis is applied.

A major challenge in the field of metagenomics is contamination. DNA from exogenous sources leads to a critical impact on results obtained from clinical samples, especially those containing low biomass (Salter et al., 2014). In our study, we observed that three bacteria (*C. acnes*, *E. coli*, and *Escherichia* unclassified) found in the negative controls were predominantly present in the patient samples. This observation allowed us to propose a strategy for the determination of contaminants. To better deal with contamination issues in CMg, the use of negative controls is mandatory and widely accepted by the scientific community. Still, the nature of the control is not. In most instances (Street et al., 2017; Ivy et al., 2018; Thoendel et al., 2018), sterile water is used, while in others, non-infected samples are used (Rodriguez et al., 2020). In this study, we selected saline solution which underwent the same handling processes as the other samples, from the surgery room to the plating on agar dishes, to capture all the situations where contaminants could be brought in. However, we sequenced two negative controls while one per surgery could have helped in identifying contaminants. As contamination is a major issue in low-biomass samples, before DNA extraction, one could also spike all samples and controls with a defined quantity of bacteria that are unrelated to the host–microbiome (Stämmler et al., 2016). This would allow absolute quantification of bacterial species and better distinction between contaminants and bacteria present in the infected tissue.

One added value of CMg over 16S rDNA sequencing is access to the antimicrobial resistance determinants (ARG and mutations associated with resistance), provided that the genome of the bacterium of interest is covered enough so that no antimicrobial resistance determinants are missed. Assessing the antimicrobial susceptibility of microorganisms found in CMg remains highly challenging and very few reports have attempted to do so (Ruppé et al., 2017; Charalampous et al., 2019). The first step is to establish rules aiming to correlate antimicrobial-resistant genetic determinants and a phenotype. For this purpose, several studies (reviewed in Ruppé et al., 2020) have shown that such correlation could be successfully achieved at a genomic level for *S. aureus*, *Enterobacterales*, and *Mycobacterium tuberculosis*. For other species, results were not as good (*Pseudomonas aeruginosa*) or no studies were conducted. Importantly, these rules were based on genomic material, hence transposing them to the metagenomic context makes a significant challenge. In this perspective, we assessed whether there was a minimal genome (as reconstructed from metagenomic data) coverage above which such inference could be achieved with confidence. We found that when the genome coverage exceeded 87.5%, a correct inference of the phenotype was consistently obtained in all cases, while below, antibiotic resistance determinants were missed in most cases. Similar experiments with a higher number of samples should be conducted in order to set a threshold with greater precision.

We acknowledge some limitations of our study. We analyzed a limited number of patients at two centers that used different bacteriological protocols for BJI samples. One center indeed used sonication before sequencing, which increases sensitivity for bacterial recovery, but also adds another step during which contamination may occur. Also, we sequenced samples at a lower sequencing depth as compared to most other clinical metagenomic studies. We previously observed that a lower depth of sequencing did not significantly modify the sensitivity for microorganism detection but lowered the sensitivity for antimicrobial resistance determinants (Ruppé et al., 2017). In addition, we were interested only in bacteriological diagnosis and did not consider other microorganisms such as yeasts, fungi, or viruses, which, however, are not commonly found in BJIs. Depending on the location of the infection, it may be appropriate to use a different protocol (e.g., no human DNA depletion by lysis of human cells or viral DNA/RNA enrichment to study eukaryotic viruses) to detect other microorganisms. Also, the Sanger method is routinely used in clinical bacteriology laboratories to sequence the 16S rDNA amplicons. As it suffers from intrinsic limitations such as the difficulty to address polymicrobial infections, the use of high-throughput sequencing instead of the Sanger method could be an alternative to shotgun metagenomics. Finally, this analytical study did not check clinical data such as antibiotic treatment or clinical outcomes of patients.

Our results support the conclusion that CMg has the potential to replace 16S rDNA sequencing (using the Sanger method) and complement conventional methods in the diagnosis of BJIs. The complementary value of CMg over culture strongly encourages its early positioning in BJI diagnosis.

## Data Availability Statement

The datasets presented in this study can be found in online repositories as BioProject, PRJNA803614.

## Ethics Statement

The studies involving human participants were reviewed and approved by the Comité d’Evaluation de l’Ethique des projets de Recherche Biomédicale (CEERB) “Paris Nord”; IRB 00006477. Written informed consent for participation was not required for this study in accordance with the national legislation and the institutional requirements.

## Author Contributions

ER conceived, designed, and supervised the study. VL, ML, MA-R, NG, F-XL, OS, LD, MD, and VI included the patients. Cd’H, SG, VL-G, NM, MD, A-CL, AN, and SF processed samples. NadG, VL, and MP performed bioinformatic analyses. Cd’H performed analyses. Cd’H and ER interpreted the data. Cd’H, ER, JS, and VL wrote the manuscript. All authors read and approved the submitted version of the manuscript.

## Conflict of Interest

GGu and GGe were employed by bioMérieux SA. SF was employed by Genoscreen. ER received consulting fees from Illumina and Pathoquest. The remaining authors declare that the research was conducted in the absence of any commercial or financial relationships that could be construed as a potential conflict of interest.

## Publisher’s Note

All claims expressed in this article are solely those of the authors and do not necessarily represent those of their affiliated organizations, or those of the publisher, the editors and the reviewers. Any product that may be evaluated in this article, or claim that may be made by its manufacturer, is not guaranteed or endorsed by the publisher.
