# Supplementary material for: Contribution of Clinical Metagenomics to the Diagnosis of Bone and Joint Infections
Source: Front Microbiol. 2022 Apr 21;13:863777. doi: 10.3389/fmicb.2022.863777 (PMC9069157; doi:10.3389/fmicb.2022.863777)
Supplement: Supplementary file 1 [file Data_Sheet_1.docx]

**Contribution of clinical metagenomics to the diagnosis of bone and joint infections**

**Supplementary Tables and Figures**

Camille d’Humières, Nadia Gaïa, Signara Gueye, Victoire de Lastours, Véronique Leflon-Guibout, Naouale Maataoui, Marion Duprillot, Marie Lecronier, Marc-Antoine Rousseau, Naura Gamany, François-Xavier Lescure, Olivia Senard, Laurène Deconinck, Marion Dollat, Valentina Isernia, Anne-Claire Le Hur, Marie Petitjean, Anissa Nazimoudine, Sylvie Le Gac, Solaya Chalal, Stéphanie Ferreira, Vladimir Lazarevic, Ghislaine Guigon, Gaspard Gervasi, Laurence Armand-Lefèvre, Jacques Schrenzel, Etienne Ruppé.

**Supplementary Figure 1**: distribution of the 99 samples according to the number of high-quality reads obtained by metagenomic sequencing.

**Supplementary Figure 2**: Bar plot of the number of samples in which contaminants (found in the two negative controls) were found (panel A) and bar plot of the 50 most frequently found bacteria in patient samples by metagenomic sequencing (panel B).

**A**

**B**

**Supplementary Figure 3**: Barplot of the number and types of antibiotic resistance genes (ARG) found in the samples.

**Supplementary Table 1**: **Detail of the culture, 16S rDNA sequencing and clinical metagenomics results.** For culture results: Q4: presence of bacteria in the last quadrant on the plate; Q3: presence of bacteria in 3 quadrants of the plate; Q2: presence of 10-100 bacteria on the plate; Q1: presence of less than 10 bacteria on the plate; P: presence in enrichment broth. TN1 and TN2 were the negative controls.

**Supplementary Table 2 legend**: Details of the antimicrobial resistance genes (ARG) found in the 27 patients with a positive culture (monomicrobial or polymicrobial): phenotypic observation and potential genotypic concordance. CMg: clinical metagenomics ; NA: not assigned; WT : wild type.
